# Association between anxiety and metabolic syndrome: An updated systematic review and meta-analysis

**DOI:** 10.3389/fpsyt.2023.1118836

**Published:** 2023-02-16

**Authors:** Shuang Ji, Yujiao Chen, Yuying Zhou, Yiting Cao, Xiao Li, Guoyong Ding, Fang Tang

**Affiliations:** ^1^Department of Neurology, Shandong Provincial Qianfoshan Hospital, Weifang Medical University and Shandong Institute of Neuroimmunology, Jinan, China; ^2^Department of Clinical Pharmacy, The First Affiliated Hospital of Shandong First Medical University & Shandong Provincial Qianfoshan Hospital, Jinan, China; ^3^Department of Clinical Pharmacy, Shandong Provincial Qianfoshan Hospital, Shandong University, Jinan, China; ^4^School of Public Health, Shandong First Medical University & Shandong Academy of Medical Sciences, Jinan, China; ^5^Center for Big Data Research in Health and Medicine, The First Affiliated Hospital of Shandong First Medical University, Jinan, China; ^6^Shandong Provincial Qianfoshan Hospital, Cheeloo College of Medicine, Shandong University, Jinan, China

**Keywords:** anxiety, metabolic syndrome, cross-sectional study, cohort study, meta-analysis

## Abstract

**Objective:**

Previous studies have demonstrated an association between anxiety and metabolic syndrome (MetS). However, the association is still controversial. This updated meta-analysis aimed to reanalyze the association between anxiety and MetS.

**Methods:**

We comprehensively searched PubMed, Embase and Web of Science for all related studies published before January 23, 2023. Observational studies that informed effect size with 95% confidence interval (CI) for the association between anxiety and MetS were included. According to heterogeneity between studies, fixed or random effects models were applied to calculate the pooled effect size. Publication bias was examined by funnel plots.

**Results:**

The research included 24 cross-sectional studies: 20 studies used MetS as the dependent variable with a pooled OR of 1.07 (95% CI: 1.01–1.13) and four studies used anxiety as the dependent variable with a pooled OR of 1.14 (95% CI: 1.07–1.23). Three cohort studies were found: two studies detected the association of baseline anxiety with the risk of MetS, one of the studies demonstrated a significant association, but a similar result was not found in another study; one study showed no significant association between baseline MetS and the risk of anxiety.

**Conclusion:**

Cross-sectional studies indicated an association between anxiety and MetS. The results from cohort studies are still inconsistent and limited. More large-scale prospective studies are needed to further reveal the causal relationship of anxiety with MetS.

## 1. Introduction

Metabolic syndrome (MetS) is an assemblage of metabolic abnormalities, characterized by obesity, increased fasting blood glucose, dyslipidemia and hypertension (1). MetS constitutes growing health problems due to it contributes to the increased risk of cardiovascular disease (CVD) and type 2 diabetes mellitus (T2DM) (2, 3). The prevalence of MetS has increased dramatically worldwide. It has reported that the prevalence of MetS among adults in the US between 2011 and 2016 was 34.7%, and the prevalence significantly increased with advancing age (4). In the past two decades, the prevalence of MetS has increased rapidly from 13.7 to 31.1% in China (5).

Previous studies indicated a mutual relationship between physical illnesses (i.e., CVD, MetS) and mental disorders (6, 7). Anxiety disorders are one of the most common mental disorders with onset usually in childhood (8, 9). The lifetime prevalence of anxiety disorders is 5–25%, and the 12 months prevalence is 3.0–19.0% (10). Previous studies indicated that the prevalence of MetS among people with anxiety ranges from 30 to 40% (11, 12). The prevalence of anxiety was approximately 10% higher among people with MetS compared with those without MetS (13). However, the association between MetS and anxiety is still inconsistent due to the differences in diagnostic criteria for anxiety and MetS, study sample size, and study design (12, 14, 15).

Our previous systematic review and meta-analysis suggested that anxiety was positively associated with MetS (16). Since our last meta-analysis, there are nine studies on the association between anxiety and MetS have been published (12, 14, 15, 17–22). Therefore, it is necessary to update the review to obtain current evidence based on the newly published research. In addition, the previous study was limited to exploring the association between anxiety and MetS, the effect of different study outcomes on this association was not explored. So, we performed an updated meta-analysis to derive a more comprehensive and reliable estimation of the association.

## 2. Materials and methods

### 2.1. Search strategy

The present study followed the Meta-analyses and Systematic Review of Observational Studies in Epidemiology (MOOSE) and Preferred Reporting Items for Systematic Reviews and Meta-Analyses (PRISMA, Supplementary Table 1) guidelines (23, 24). We performed systematic literature searches in PubMed, Embase and Web of Science for all related studies published before January 23, 2023. Two authors (SJ and YCH) independently conducted the search using MeSH terms and free terms for “anxiety” and “metabolic syndrome.” An example search strategy for the three databases is listed in Supplementary Table 2. All eligible studies were limited to those published in English. Manual searches of the reference lists of the included studies were performed.

### 2.2. Study selection

After we searched the literature, titles, abstracts and full-text articles were reviewed by two independent authors (SJ and YCH) to identify studies that met the inclusion criteria. Any discrepancies were resolved by consensus. Articles were considered for inclusion according to the following criteria: (1) observational study design (including cross-sectional study, cohort study or case-control study); (2) studies that measured MetS using International Diabetes Federation (IDF), National Cholesterol Education Program-Adult Treatment Panel III (NCEP-ATP III) or World Health Organization (WHO) criteria; and (3) studies presenting the effect size of the association between anxiety and MetS [odds ratio (OR), relative risk (RR), or hazard ratio (HR) with 95% confidence interval (CI)] or sufficient data to estimate it. Reviews, conference abstracts, letters to editors and unpublished studies not meeting the criteria above were excluded. Studies with low quality scores were excluded from further analysis. If more than one eligible publication was conducted within the same population, only the most relevant study was chosen.

### 2.3. Data extraction and quality assessment

The following general characteristics of the eligible studies were collected by two authors: study characteristics (first author, country, year of publication, study design etc.), follow-up time for cohort studies, population, sample size, age, sex, anxiety measurement, definition of MetS, statistical results (unadjusted or adjusted OR, RR, or HR with its 95% CI) and adjustment for potential confounding factors. The Agency for Healthcare Research and Quality (AHRQ) and Newcastle-Ottawa scale (NOS) criteria were used to evaluate the quality of the included cross-sectional studies and cohort studies, respectively. The AHRQ criteria was scored as follows: low quality = 0–3; moderate quality = 4–7; high quality = 8–11 (25). According to the NOS, the included studies were defined as low quality (1–3 stars), moderate quality (4–6 stars) and high quality (7–9 stars) (26). Methodologically, low quality studies tend to exaggerate the overall estimate and may lead to incorrect inferences (27). Therefore, we removed the studies with low quality. Two independent authors (SJ and YCH) performed the data extraction and quality assessment. Any disagreement was resolved through negotiation until an agreement was reached.

### 2.4. Statistical analysis

The included studies with effect sizes (ORs, RRs, or HRs) for the association between anxiety disorders and MetS were synthesized into pooled ORs and RRs with corresponding 95% CIs. Heterogeneity among the included studies was detected with the *Q* test and the *I*^2^ statistic. A random or fixed effects model was used depending on heterogeneity among studies. *I*^2^ > 50% indicates heterogeneity among studies, and random effects model was adopted as the pooling method; otherwise, we used a fixed effects model to pool effect sizes (28). Subgroup analyses were performed to explore the heterogeneity across studies based on anxiety assessment, MetS diagnosis criteria, country, sample size, and study quality. Sensitivity analyses removing one individual study each time were investigated to evaluate the stability of the pooled results. In addition, publication bias was evaluated by funnel plot symmetry, Egger’s and Begg’s tests. Data analyses were performed using STATA/MP 16.0. In the bilateral situation, *P* < 0.05 was considered to be statistically significant. However, *P* < 0.1 illustrated heterogeneity among studies in the *Q* test (29).

## 3. Results

### 3.1. Characteristics of the included studies

**FIGURE 1:**
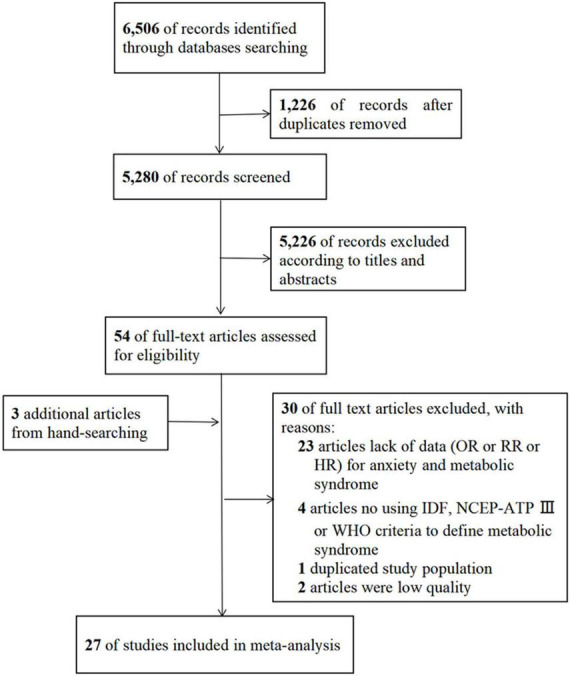
Flowchart of the literature search and study selection. HR, hazard ratio; IDF, International Diabetes Federation; NCEP-ATP III, National Cholesterol Education Program-Adult Treatment Panel III; OR, odds ratio; RR, relative risk; WHO, World Health Organization.

A flowchart of the literature screening process is presented in Figure 1. We searched three additional literatures manually. A total of 27 studies ultimately met the inclusion criteria (12, 14, 15, 17–22, 30–47). There were six high quality studies and 21 moderate quality studies. Information about the quality assessment of eligible studies can be obtained from Supplementary Tables 4, 5.

The characteristics of the 24 cross-sectional studies are presented in Supplementary Table 3. Twenty studies reported MetS as the dependent variable (12, 14, 15, 17, 18, 30–37, 39, 40, 43–47) and four studies reported anxiety as the dependent variable (19, 20, 22, 41). Twenty-four studies used two methods to diagnose anxiety, seven studies used structured diagnostic interviews, and 17 studies used self-report symptom scales. NCEP-ATP III (*n* = 19) and IDF (*n* = 5) were used to define MetS. The 24 studies were conducted in fifteen different countries (i.e., America, Brazil, Finland, China, France, Germany, Greece, Iran, Italy, Japan, Lithuania, Netherlands, Norway, Switzerland, and Thailand).

Supplementary Table 3 displays the characteristics of the cohort studies. Three cohort studies were included (21, 38, 42), and two reported the association of baseline anxiety status with the risk of MetS. In contrast, another study reported that baseline MetS status predicted the risk of anxiety. The follow-up duration in cohort studies varied from 1 to 15 years.

### 3.2. Association of anxiety with metabolic syndrome based on cross-sectional studies

**FIGURE 2:**
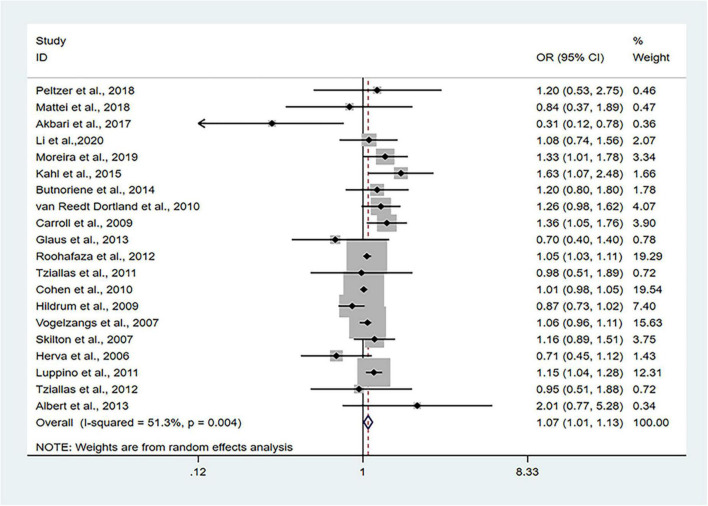
Forest plots of the association of anxiety with MetS among cross-sectional studies with MetS as the dependent variable. CI, confidence interval; MetS, metabolic syndrome; OR, odds ratio.

The 24 cross-sectional studies included in the present analysis comprised 80,466 subjects. We calculated the pooled OR with MetS as the dependent variable using the random effects model given that considerable heterogeneity was noted (*I*^2^ = 51.3%, *P_*Q*_* = 0.004). The pooled results of random effects analysis showed that patients with anxiety had a higher risk of MetS than people without anxiety, with a pooled OR of 1.07 (95% CI: 1.01–1.13) (Figure 2).

To investigate the factors affecting heterogeneity, subgroup analyses according to anxiety assessment, MetS diagnosis criteria, country, sample size and study quality were performed (Table 1). On the basis of anxiety assessment, we found that using structured clinical interviews tended to report a stronger association than using self-reported symptom scales. The pooled ORs of the two assessment methods were 1.29 (95% CI: 1.13–1.48, *I*^2^ = 4.02%) and 1.04 (95% CI: 0.99–1.09, *I*^2^ = 43.60%), respectively. Based on MetS diagnostic criteria, the NCEP-ATP III criteria expressed a stronger association between anxiety and MetS (OR: 1.09, 95% CI: 1.03–1.16, *I*^2^ = 51.33%). The IDF criteria presented a negative but non-significant association (OR: 0.89, 95% CI: 0.76–1.04, *I*^2^ = 0%). The *P*-values of the chi-square test among the two subgroups above indicated statistical significance (Table 1). Differences in the diagnosis of anxiety and MetS might be the source of heterogeneity. The heterogeneity in other subgroups was not significant, which indicated that these factors were not the source of heterogeneity.

**TABLE 1:** Results of subgroup analysis stratified by anxiety assessment, metabolic syndrome (MetS) diagnosis criteria, country, sample size and study quality.

Subgroups	No. of studies	OR (95% CI)	*I*^2^ (%)	*P-*value for heterogeneity	Chi-square test *P*-value
**Anxiety assessment**	–	–	-	-	<0.01
Self-report symptom scales	14	1.04 (0.99–1.09)	43.60	0.04	-
Structured clinical interviews	6	1.29 (1.13–1.48)	4.02	0.39	-
**MetS diagnosis criteria**	–	–	-	-	0.02
NCEP-ATP III	16	1.09 (1.03–1.16)	51.33	<0.01	-
IDF	4	0.89 (0.76–1.04)	0.00	0.87	-
**Country**	–	–	-	-	0.88
Developing	6	1.09 (0.90–1.31)	48.46	0.08	-
Developed	14	1.07 (0.99–1.10)	54.45	0.01	-
**Sample size**	–	–	-	-	0.96
<1000	8	1.07 (0.83–1.38)	46.13	0.07	-
≥1000	12	1.07 (1.00–1.16)	56.84	0.01	-
**Study quality**	–	–	-	-	0.46
High	5	1.09 (1.03–1.16)	0.00	0.66	-
Moderate	15	1.05 (0.98–1.14)	57.91	<0.01	-

CI, confidence interval; IDF, international diabetes federation; MetS, metabolic syndrome; No., number; NCEP-ATP III, national cholesterol education program-adult treatment panel III; OR, odds ratio.

**FIGURE 3:**
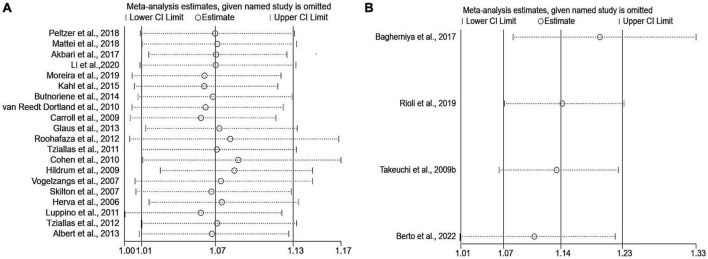
Sensitivity analyses of the association of anxiety with MetS. **(A)** Metabolic syndrome as the dependent variable; **(B)** anxiety as the dependent variable. MetS: metabolic syndrome.

**FIGURE 4:**
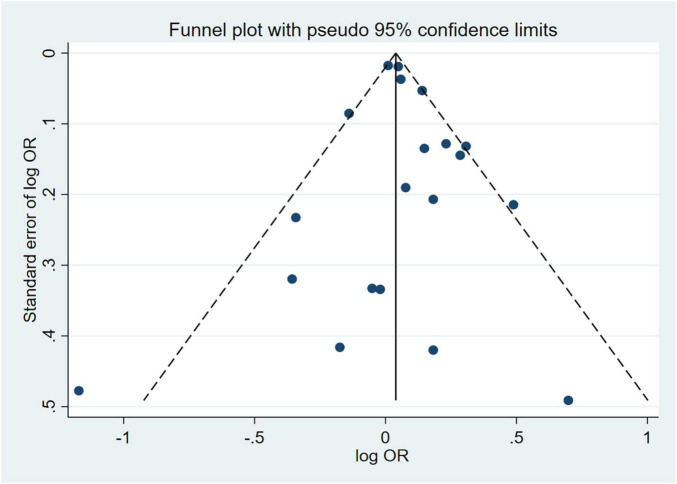
Funnel plot of effect estimates for 20 cross-sectional studies with MetS as the dependent variable assessing the association between anxiety and MetS (*P* = 0.51 by Egger’s test and *P* = 0.50 by Begg’s test). MetS, metabolic syndrome; OR, odds ratio.

Sensitivity analyses that eliminated each study in turn were performed to examine whether individual studies affected the pooled results. Sensitivity analyses illustrated that there was no substantial modification in the results after removing any of the studies (varied from 1.00 to 1.17) (Figure 3A). Results of Begg’s test (*P* = 0.50) and Egger’s test (*P* = 0.51) did not observe publication bias. Nor did the funnel plot exhibit obvious asymmetry (Figure 4), suggesting no publication bias in our analysis.

**FIGURE 5:**
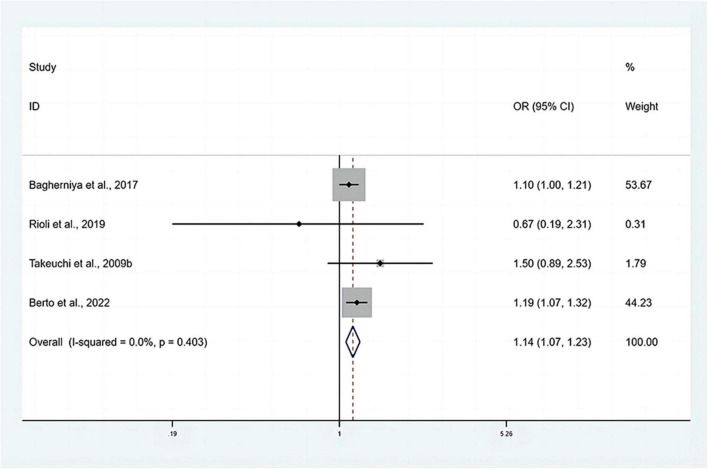
Forest plots of the association of anxiety with MetS among cross-sectional studies with anxiety as the dependent variable. CI, confidence interval; MetS, metabolic syndrome; OR, odds ratio.

The pooled OR with anxiety as the dependent variable is presented in Figure 5. The results showed that individuals with MetS expressed significantly higher risks of anxiety than people without MetS, with no heterogeneity (OR: 1.14, 95% CI: 1.07–1.23; *I*^2^ = 0.0%). The sensitivity analysis is presented in Figure 3B, and the results showed that no individual study significantly influenced the pooled OR.

### 3.3. Association of anxiety with metabolic syndrome based on cohort studies

Two cohort studies explored the association between baseline anxiety status and future risk of MetS. One prospective cohort study, which included 432 participants with a 15 years follow-up, used IDF, NCEP-ATP III, and WHO criteria to diagnose MetS. The assessment of anxiety was performed using the Spielberger Trait Anxiety Questionnaire (38). The adjusted RRs were 1.04 (95% CI: 0.87–1.25), 1.07 (95% CI: 0.87–1.32), and 1.08 (95% CI: 0.76–1.54) for the IDF, NCEP-ATP III and WHO criteria, respectively. The other study was a retrospective cohort study designed by Lubas et al. (21) with an average of 3.9 years of follow-up. A total of 3,267 participants were included in this study, 809 of whom were diagnosed with MetS according to the NCEP-ATP III criteria. Anxiety disorder was evaluated using the Brief Symptom Inventory-18 (BSI-18). The adjusted RR was 1.34 (95% CI: 1.12–1.59). The quality scores of the two studies were both of seven (Supplementary Table 5), and the characteristics of the two studies are presented in Supplementary Table 3.

One cohort study investigated the association between baseline MetS status and the future risk of anxiety with a total sample size of 956. The characteristics of the study are shown in Supplementary Table 3. The follow-up time of this study was 1 year, and the quality score was six (Supplementary Table 5) (42). MetS was assessed according to the IDF criteria. The profile of mood states (POMS) was used to define anxiety. The RR of the association was 0.7 (95% CI: 0.35–1.41).

## 4. Discussion

This updated systematic review and meta-analysis has consistently confirmed the association between anxiety and MetS using the data from cross-sectional studies with anxiety and MetS as dependent variables, respectively. Subgroup analysis including anxiety assessment, MetS diagnosis criteria, country, study sample size and study quality showed that the diagnostic criteria for anxiety and MetS could influence the strength of the association.

The final pooled OR based on the cross-sectional studies was 1.07 (95% CI: 1.01–1.13) for MetS as the dependent variable and 1.14 (95% CI: 1.07–1.23) for anxiety as the dependent variable. Our findings are consistent with other studies included in this study that prior anxiety increased the risk of MetS (12, 31, 36, 37, 39). The subgroup analyses according to different diagnostic criteria for anxiety suggested that the association was stronger for anxiety assessed by structured clinical interviews compared with the self-reported symptom scale. A previous study suggested that anxiety, which could coexist with one or more mental disorders, may have the same symptoms as other psychiatric disorders (48). This may result in the inclusion of individuals who do not meet the formal criteria for the Diagnostic and Statistical Manual of Mental Disorders (DSM) diagnosis. The accuracy of structured clinical interviews in diagnosing anxiety may explain the higher association between anxiety and metabolic syndrome.

We observed that the result of the NCEP-ATP III criteria was similar to the main results. The IDF criteria indicated a non-significant association between anxiety and MetS. Considering that the IDF criteria focused on abdominal obesity and its mandatory obesity thresholds and diagnostic segmentation points of metabolic abnormalities are lower than those recommended by NCEP-ATP III criteria, it is expected that the IDF criteria would detect a higher proportion of patients with MetS (49), which may explain the difference between the two diagnostic criteria. We also observed that the result of studies with sample sizes greater than 1,000 was similar to the main results. However, the result from studies with a small sample size was insignificant. In the subgroup analyses of anxiety and MetS risk according to study quality, we observed that the increased risk reported in high quality studies was similar to that reported in the overall analysis, while a non-significant increase in risk was observed for moderate quality studies. However, the difference between high and moderate quality was insignificant, indicating that quality was not the main source of the overall heterogeneity.

According to the pooled results of the cross-sectional studies included, the causality of this association could not be assessed. If anxiety occurs earlier than MetS, the following pathophysiological mechanisms of anxiety may contribute to MetS. First, anxiety is correlated with sympathetic nervous system excitation, which will lead to the contraction of systemic arterioles and increased peripheral vascular resistance that cause the elevation of blood pressure. Increased secretion of catecholamines from the adrenal glands could also increase blood pressure (50–52). Second, the evidence suggested that anxiety could enhance the excitability of the hypothalamic-pituitary-adrenocortical (HPA) axis, which will increase the secretion of cortisol (53–55). The chronically high concentration of cortisol inhibits the pro-glycemic uptake function of insulin and causes the increase of blood glucose. In addition, cortisol has a strong aggregation effect on adipose tissue, and the abnormal increase of cortisol may cause visceral fat deposition, leading to obesity and dyslipidemia (56, 57). Third, anxiety could promote the inflammatory response of patients, and some inflammatory factors, such as interleukin-6 (IL-6) and tumor necrosis factor-α (TNF-α), are risk factors for insulin resistance, which could contribute to insulin resistance, thereby promoting the prevalence of MetS (58, 59).

If MetS occurs prior to anxiety, the pathophysiologic mechanisms may be attributed to insulin resistance. Insulin, as a key hormone in regulating carbohydrate metabolism, not only promotes glucose uptake and glucose oxidation but is also essential for maintaining normal brain function (60). It has been suggested that total cerebral blood flow which maintains the normal function of brain tissue was 15% lower in patients with MetS (61). There is evidence indicated that peripheral insulin resistance could lead to down-regulation of insulin receptor activity in brain tissue and decrease the level of insulin entering brain tissue, which may result in low metabolism of brain tissue and abnormalities of different functional areas of the brain (62, 63). The frontal lobe, as an important functional area of emotion regulation, is often affected and ultimately contributes to the occurrence of anxiety (64). Furthermore, insulin resistance in brain tissue may cause structural and functional damage in the hippocampus, resulting in hyperfunction of the HPA axis (65). The hyperfunction of the HPA axis contributes to the disturbance of cortisol rhythm and the imbalance of salt and glucocorticoids in the brain, which leads to abnormal levels of 5-hydroxytryptamine (5-HT). Dysfunction of the 5-HT system also promotes the occurrence of anxiety (66). Finally, prospective studies have demonstrated that insulin resistance and impaired glucose tolerance are related to somnipathy, which is one of the causes of anxiety (67–69).

The association between anxiety and MetS could also be attributed to other factors. Emotional eating is an emotional response led by negative stimuli, such as feeling stressed or anxious (70). In the context of acute psychosocial stress, cortisol levels which may influence eating behavior were higher in patients with anxiety, and high cortisol levels could contribute to binge eating (71).

The findings of this meta-analysis are of important clinical significance. Anxiety has been documented to contribute to the risk of CVD in initially healthy people (72, 73). MetS is not only a risk factor for T2DM and CVD but also related to anxiety (19, 74). For patients with anxiety, the risk factors for MetS should be routinely checked. Proper lifestyle modification and pharmaceutical therapy should be proposed if patients are at high risk of adiposity, hyperglycemia and CVD. In addition, for patients with MetS, appropriate attention should be given to their psychological condition rather than limiting the treatment to physical disease. For patients already suffering from anxiety, psychological treatment or pharmacological treatment could be used to reduce mental and physical symptoms, and improve their social and interpersonal relationships (75).

Previous studies have demonstrated that psychotropic medications could impact cardiovascular health and increase MetS risk in psychiatric patients (76, 77). It has been widely reported that second-generation antipsychotics (SGAs), including antidepressants and anxiolytics, are associated with weight gain, lipid disorder and dysregulation of glucose (78). Antipsychotics also have effects on the central nervous system, inflammatory response, and cellular metabolism (79). Therefore, providing medication guidance for patients with anxiety, which could minimize the risk of MetS and prevent iatrogenic MetS, is of significant interest.

This meta-analysis has several strengths. To our knowledge, this is the first meta-analysis that evaluates the association between anxiety and MetS using data from both cross-sectional and cohort studies with anxiety and MetS as dependent variables, respectively. Moreover, we conducted comprehensive subgroup analyses to reveal the heterogeneity and indicated that the diagnostic criteria for anxiety and MetS could influence the strength of the association. However, the limitations of this study merit consideration. First, the meta-analysis involved mainly cross-sectional studies, the cause-and-effect relationship of anxiety with MetS cannot be proven. Second, studies with anxiety as the dependent variable were limited, more studies are recommended to assess the impact of MetS on anxiety. Third, our study focused on the overall association between anxiety and MetS, further study could concentrate on the association between specific MetS components and anxiety.

## 5. Conclusion

This updated systematic review and meta-analysis added the evidence of the association between anxiety and MetS. Psychologists treating individuals with anxiety should detect changes in metabolic components, and physicians dealing with MetS patients should be aware of their possible predisposition to anxiety. As most of the studies included in the current study were cross-sectional studies, no conclusions on causal inference could be reached. Therefore, more prospective studies, particularly long-term follow-up and large-scale cohort studies, are required to explain the causal relationship of anxiety with MetS.

## Author contributions

FT and GD conceived the study protocol. SJ and YCh conducted the literature search and selection, data extraction and analysis, and drafted the manuscript. All authors participated in the discussions and interpretation of the results and approved the final version of the manuscript.
