# Supplementary material for: Association between anxiety and metabolic syndrome: An updated systematic review and meta-analysis
Source: Front Psychiatry. 2023 Feb 16;14:1118836. doi: 10.3389/fpsyt.2023.1118836 (PMC9978147; doi:10.3389/fpsyt.2023.1118836)
Supplement: Supplementary file 1 [file Table_1.docx]

**Supplementary Material**

**Contents**

Supplementary Table 1. PRISMA checklist. …………………………...…………...1-2

Supplementary Table 2. Search strategy for three peer-reviewed databases. ……...… 3

Supplementary Table 3. Characteristics of included studies in the meta-analysis. …………………...................…………………………………….………. 4-9

Supplementary Table 4. Quality assessment of cross-sectional studies included in the meta-analysis...........................................................................................................10-11

Supplementary Table 5. Quality assessment of cohort studies included in the meta-analysis.........................................................................................................................12

**Supplementary Table 1.** PRISMA checklist.

| **Section/topic** | **#** | **Checklist item** | **Reported on page #** |
| --- | --- | --- | --- |
| **TITLE** |  |  |  |
| Title | 1 | Identify the report as a systematic review, meta-analysis, or both. | 1 |
| **ABSTRACT** |  |  |  |
| Structured summary | 2 | Provide a structured summary including, as applicable: background; objectives; data sources; study eligibility criteria, participants, and interventions; study appraisal and synthesis methods; results; limitations; conclusions and implications of key findings; systematic review registration number. | 1 |
| **INTRODUCTION** |  |  |  |
| Rationale | 3 | Describe the rationale for the review in the context of what is already known. | 2 |
| Objectives | 4 | Provide an explicit statement of questions being addressed with reference to participants, interventions, comparisons, outcomes, and study design (PICOS). | 2 |
| **METHODS** |  |  |  |
| Protocol and registration | 5 | Indicate if a review protocol exists, if and where it can be accessed (e.g., Web address), and, if available, provide registration information including registration number. | NA |
| Eligibility criteria | 6 | Specify study characteristics (e.g., PICOS, length of follow-up) and report characteristics (e.g., years considered, language, publication status) used as criteria for eligibility, giving rationale. | 2 |
| Information sources | 7 | Describe all information sources (e.g., databases with dates of coverage, contact with study authors to identify additional studies) in the search and date last searched. | 2 |
| Search | 8 | Present full electronic search strategy for at least one database, including any limits used, such that it could be repeated. | 2 |
| Study selection | 9 | State the process for selecting studies (i.e., screening, eligibility, included in systematic review, and, if applicable, included in the meta-analysis). | 2 |
| Data collection process | 10 | Describe method of data extraction from reports (e.g., piloted forms, independently, in duplicate) and any processes for obtaining and confirming data from investigators. | 2 |
| Data items | 11 | List and define all variables for which data were sought (e.g., PICOS, funding sources) and any assumptions and simplifications made. | 2 |
| Risk of bias in individual studies | 12 | Describe methods used for assessing risk of bias of individual studies (including specification of whether this was done at the study or outcome level), and how this information is to be used in any data synthesis. | NA |
| Summary measures | 13 | State the principal summary measures (e.g., risk ratio, difference in means). | 2 |
| Synthesis of results | 14 | Describe the methods of handling data and combining results of studies, if done, including measures of consistency (e.g., *I^2^*) for each meta-analysis. | 2 |
| Risk of bias across studies | 15 | Specify any assessment of risk of bias that may affect the cumulative evidence (e.g., publication bias, selective reporting within studies). | 2 |
| Additional analyses | 16 | Describe methods of additional analyses (e.g., sensitivity or subgroup analyses, meta-regression), if done, indicating which were pre-specified. | 2 |
| **RESULTS** |  |  |  |
| Study selection | 17 | Give numbers of studies screened, assessed for eligibility, and included in the review, with reasons for exclusions at each stage, ideally with a flow diagram. | 3 |
| Study characteristics | 18 | For each study, present characteristics for which data were extracted (e.g., study size, PICOS, follow-up period) and provide the citations. | 3 |
| Risk of bias within studies | 19 | Present data on risk of bias of each study and, if available, any outcome level assessment (see item 12). | NA |
| Results of individual studies | 20 | For all outcomes considered (benefits or harms), present, for each study: (a) simple summary data for each intervention group (b) effect estimates and confidence intervals, ideally with a forest plot. | 3 |
| Synthesis of results | 21 | Present results of each meta-analysis done, including confidence intervals and measures of consistency. | 3 |
| Risk of bias across studies | 22 | Present results of any assessment of risk of bias across studies (see Item 15). | 3 |
| Additional analysis | 23 | Give results of additional analyses, if done (e.g., sensitivity or subgroup analyses, meta-regression [see Item 16]). | 3 |
| **DISCUSSION** |  |  |  |
| Summary of evidence | 24 | Summarize the main findings including the strength of evidence for each main outcome; consider their relevance to key groups (e.g., healthcare providers, users, and policy makers). | 5-7 |
| Limitations | 25 | Discuss limitations at study and outcome level (e.g., risk of bias), and at review-level (e.g., incomplete retrieval of identified research, reporting bias). | 7 |
| Conclusions | 26 | Provide a general interpretation of the results in the context of other evidence, and implications for future research. | 7 |
| **FUNDING** |  |  |  |
| Funding | 27 | Describe sources of funding for the systematic review and other support (e.g., supply of data); role of funders for the systematic review. | 7 |

Abbreviations: NA: not available.

**Supplementary Table 2.** Search strategy for three peer-reviewed databases.

| Database | Step | Searching strategy | Number of articles |
| --- | --- | --- | --- |
| PubMed | #1 | "anxiety"[MeSH Terms] OR "anxiety"[Title/Abstract] OR "tension"[Title/Abstract] OR "panic"[Title/Abstract] OR "worry"[Title/Abstract] OR "phobic anxiety"[Title/Abstract] OR "phobia"[Title/Abstract] OR "posttraumatic stress disorder"[Title/Abstract] | 396,053 |
|  | #2 | "Metabolic Syndrome"[MeSH Terms] OR "metabolic syndromes"[Title/Abstract] OR "Metabolic Syndrome"[Title/Abstract] OR "syndrome x"[Title/Abstract] OR "dysmetabolic syndrome x"[Title/Abstract] OR "cardiovascular syndrome"[Title/Abstract] OR "insulin resistance"[Title/Abstract] OR "insulin resistance syndrome x"[Title/Abstract] | 141,161 |
|  | #3 | inception to 2023/01 | -- |
|  | #4 | Language: English | -- |
|  | #5 | #1**AND**#2**AND**#3**AND#**4 | **985** |
| Embase | #1 | 'metabolic syndrome X '/exp OR 'metabolic syndromes':ab,ti OR 'metabolic syndrome':ab,ti OR 'syndrome X ':ab,ti OR 'dysmetabolic syndrome X ':ab,ti OR 'cardiovascular syndrome':ab,ti OR 'insulin resistance':ab,ti OR 'insulin resistance syndrome X ':ab,ti | 213,452 |
|  | #2 | 'anxiety'/exp OR anxiety:ab,ti OR tension:ab,ti OR panic:ab,ti OR worry:ab,ti OR 'phobic anxiety' :ab,ti OR phobia:ab,ti OR 'posttraumatic stress disorder':ab,ti | 557,430 |
|  | #3 | Inception to 2023/01 | -- |
|  | #4 | Language: English | -- |
|  | #5 | #1 **AND** #2 **AND** #3**AND**#4 | **2,294** |
| Web of Science | #1 | TS = (anxiety OR tension OR panic OR worry OR phobic anxiety OR phobia OR posttraumatic stress disorder) | 602,618 |
|  | #2 | TS = ('metabolic syndromes' OR 'metabolic syndrome' OR 'syndrome X ' OR 'dysmetabolic syndrome X ' OR 'cardiovascular syndrome' OR 'insulin resistance' OR 'insulin resistance syndrome X ') | [330,606](http://www--webofscience--com--https.webofknowledge.wfyxy.qfclo.com:50002/wos/woscc/summary/92a267ff-f869-45ce-a03d-28532f712673-0b5eec63/relevance/1) |
|  | #3 | Inception to 2023/01 | -- |
|  | #4 | Language: (English) AND Document types: (Article) | -- |
|  | #5 | #1**AND** #2**AND**#3**AND**#4 | **3,227** |

**Supplementary Table 3.** Characteristics of included studies in the meta-analysis.

| **Author, year** | **Location** | **Name of the study or source of participants** | **Assessment of anxiety** | **Definition of MetS** | **Size of population** | **Proportion of men**  **n (%)** | **Age of population**  **(range / mean ± SD)** | **Dependent variable** | **OR (95% CI)** | **Confounding factors** | | |
| --- | --- | --- | --- | --- | --- | --- | --- | --- | --- | --- | --- | --- |
| **Cross-sectional studies** | | | | | | | | | | | |  |
| Peltzer & Pengpid, 2018 | Thailand | Temple members | GAD-7 | IDF | 401 | 107 (26.7) | 35 - 65 | MetS | 1.20 (0.53 - 2.75) | Age, educational level, current smoker, hazardous or harmful drinker | | |
| Mattei et al., 2018 | Italy | Italian primary care patients | HADS-A | NCEP-ATP III / IDF | 129 | 55 (43.0) | 61 ± 12 | MetS | 0.84 (0.37 - 1.89) | Age, work condition, education, living with family, smoking, alcohol intake and medical comorbidity | | |
| Akbari et al., 2017 | Iran | Isfahan Cohort Study | HADS-A | NCEP-ATP III | 470 | 236 (50.2) | 55.7 ± 9.3 | MetS | 0.31 (0.12 - 0.78) | Age, gender, BMI, smoking and age‑gender interaction | | |
| Moreira et al., 2019 | Brazil | NR | DSM-IV | NCEP-ATP III | 1,023 | 418 (40.9) | 21 - 32 | MetS | 1.33 (1.01 - 1.78) | Age, ethnicity, socioeconomic status, schooling, employed, smoking, psychoactive drug use, alcohol use and excess weight | | |
| R. cen Li et al., 2020 | China | Health Promotion Center of West China Hospital | SAS | NCEP-ATP III | 19,006 | 10,788 (56.8) | 42.92 ± 9.08 | MetS | 1.08 (0.74 - 1.56) | Sex, age, alcohol and cigarette consumption | | |
| Bagherniya et al., 2017 | Iran | MASHAD study | BAI | NCEP-ATP III / IDF | 9,829 | 3,943 (40.1) | 35 - 65 | Anxiety | 1.10 (1.00 - 1.21) | Age, gender, job status, marital status, physical activity, total cholesterol, and body mass index | | |
| Rioli et al., 2019 | Italy | Italian Cross-Sectional Study | HADS-A | NCEP-ATP III / IDF | 54 | 27 (50) | 44 - 82 | Anxiety | 0.67 (0.19 - 2.31) | Age, body mass index, total cholesterol, smoke, alcohol and sedentary lifestyle | | |
| Kahl et al., 2015 | Germany | FINDRISK score | DSM-IV | NCEP-ATP III | 150 | 85 (56.7) | 56.9 ± 8.1 | MetS | 1.63 (1.07 - 2.48) | Weight, height, physical activity, alcohol intake, smoking, lifetime depression and current major depressive disorder | | |
| Butnoriene et al., 2014 | Lithuania | NR | DSM-IV | NCEP-ATP III | 1,115 | 562 (50.4) | 62.0 ± 9.6 | MetS | 1.20 (0.80 - 1.80) | Age, gender, education, residence, marital status, social status, smoking, physical activity, alcohol consumption, history of myocardial infarction, history of stroke and current major depressive episode | | |
| Roohafza et al., 2012 | Iran | Isfahan Cohort Study | HADS-A | NCEP-ATPIII | 468 | 217 (46.4) | 56.3 ± 9.8 | MetS | 1.05 (1.03 - 1.11) | Age, sex, smoking, body mass index and total cholesterol | | |
| Tziallas et al., 2011 | Greece | NR | HADS-A | IDF | 359 | 174 (48.5) | 54.6 ± 12.1 | MetS | 0.98 (0.51 - 1.89) | Age, gender, marital status, educational status, employment status, household income, smoking status, depressive symptoms above cut-off and type D personality | | |
| Cohen et al., 2010 | US | Heart and Soul Study | HADS-A | NCEP-ATP III | 1,024 | 840 (82.0) | 66.6 ± 10.6 | MetS | 1.01 (0.98 - 1.05) | Age, sex, race/ethnicity, income, education, physical activity, smoking, regular alcohol use and body mass index | | |
| Van Reedt Dortland et al., 2010 | Netherlands | Netherlands Study of Depression and Anxiety | DSM-IV | NCEP-ATP III | 1,846 | 655 (35.5) | 18 - 65 | MetS | 1.26 (0.98 - 1.62) | Age, sex, years of education, clinic site and oral contraceptive use, smoking status, alcohol use and physical activity | | |
| Carroll et al., 2009 | US | Vietnam Experience Study | DSM-III | NCEP-ATP III | 4,256 | 4,256 (100) | 31.1 - 49.0 | MetS | 1.36 (1.05 - 1.76) | Age, place of service, ethnicity, marital status, alcohol consumption, smoking, IQ, household income and education grade | | |
| Hildrum et al., 2009 | Norway | Nord-Trøndelag Health Study | HADS-A | IDF | 9,571 | 4,823 (50.4) | 47.7 ± 16.8 | MetS | 0.87 (0.73 - 1.02) | Age, gender, education, physical activity, smoking and pulse rate | | |
| Takeuchi et al., 2009 | Japan | NR | POMS | IDF | 1,215 | 1,215 (100) | 42.5 ± 10.3 | Anxiety | 1.50 (0.89 - 2.53) | Age, prior history of cardiovascular disease, type 2 diabetes, lifestyle habits (smoking, alcohol consumption, exercise, sleep) and job situation (demands, control, support and over commitment) | | |
| Vogelzangs et al., 2007 | US | Aging and Body Composition study | Hopkins Symptom Checklist anxiety subscale | NCEP-ATP III | 2,917 | 1,415 (48.5) | 73.6 ± 2.9 | MetS | 1.06 (0.96 - 1.11) | Age, sex, race, education, income, smoking, alcohol use and physical activity | | |
| Skilton et al., 2007 | France | NR | HADS-A | NCEP-ATP III | 1,598 | 1,006 (62.9) | 51.8 ± 9.8 | MetS | 1.16 (0.89 - 1.51) | Age, prior cardiovascular disease, employment status, marital status, smoking status, dietary score, physical activity and body mass index | | |
| Herva et al., 2006 | Finland | Northern Finland Birth Cohort Study | Hopkins Symptom Checklist anxiety subscale | NCEP-ATP III | 5,698 | 2,832 (49.7) | NR | MetS | 0.71 (0.45 - 1.12) | Gender, smoking, alcohol consumption, marital status, level of education and physical activity | | |
| Luppino et al., 2011 | Netherlands | Study of Depression and Anxiety | MASQ | NCEP-ATP III | 2,433 | 805 (33.1) | 42.3 ± 13.1 | MetS | 1.15 (1.04 - 1.28) | Age, sex, years of education, smoking status, alcohol use and physical activity | | |
| Albert et al., 2013 | Italy | NR | Hamilton Anxiety Rating Scale | NCEP-ATP III | 104 | 62 (59.6) | 35.9 ± 14.0 | MetS | 2.01 (0.77 - 5.28) | Unadjusted OR | | |
| Tziallas et al., 2012 | Greece | Primary Care Family Screening Program | HADS-A | IDF | 359 | 174 (48.5) | 54.9 ± 12.0 | MetS | 0.95 (0.51 - 1.88) | Age, sex, marital status, education levels, working status, household income and depression | | |
| Glaus et al., 2013 | Switzerland | The CoLaus study | DSM-IV | NCEP-ATP III | 3,716 | 826 (49.1) | 35 - 66 | MetS | 0.70 (0.40 - 1.40) | Age, sex, Socio-economic Status, physical inactivity, smoking, Bipolar-I and II, dysthymia, major depressive disorder and panic disorder | | |
| Berto et al., 2022 | Brazil | Brazilian Longitudinal Study of Adult Health | CIS-R | NCEP- ATP III | 12,725 | 5739 (45.1) | 51.8±8.9 | Anxiety | 1.19 (1.07–1.32) | Age, sex, university degree, ethnicity, smoking, physical activity, alcohol abuse, and use of anti-depressants. | | |
| **Cohort studies** | | | | | | | | | | |  |  |
| Lubas et al., 2021 | America | SJLIFE | BSI-18 | NCEP-ATP III | 3,267 | 1,639 (51.6) | 31.1 ± 8.4 | MetS | 1.34 (1.12 - 1.59) | Occupational status, marital status and income | | |
| Räikköonen et al., 2007 | Finland | Healthy Women Study | Spielberger Trait Anxiety Questionnaire | WHO criterion / NCEP-ATP III / IDF | 432 | 0 (0) | NR | MetS | 1.08 (0.76 - 1.54) ^a^  1.07 (0.87 - 1.32) ^b^  1.04 (0.87 - 1.25) ^c^ | Age, physical activity, alcohol consumption, current smoking status, use of hormone replacement therapy and level of education | | |
| Takeuchi et al., 2009 | Japan | NR | POMS | IDF | 956 | 956 (100) | 42.7 ± 10.2 | Anxiety | 0.70 (0.35 - 1.41) | Age, a history of cardiovascular disease, a history of type 2 diabetes, smoking status, alcohol consumption, exercise, sleep patterns and work-related factors (job demand, job control and social support as per the Job Content Questionnaire) | | |

Abbreviations: BAI: Beck Anxiety Inventory; BMI: body mass index; BSI-18: Brief Symptom Inventory-18; CI: confidence interval; CIS-R: Clinical Interview Schedule-Revised; DSM-III, DSM-IV: Diagnostic and Statistical Manual of Mental Disorders, 3rd and 4th editions; FINDRISK: Finnish Diabetes Risk Score; GAD-7: Generalized Anxiety Disorder 7-Item; GHQ-A: General Health Questionnaire subscale for anxiety; HADS-A: Hospital Anxiety and Depression Scale for anxiety; HAM-A: Hamilton Anxiety Rating Scale; IDF: International Diabetes Federation; IQ: intelligence quotient; MASHED: Mashhad stroke and heart atherosclerotic disorder; MASQ: Mood and Anxiety Symptom Questionnaire; MetS: metabolic syndrome; NCEP-ATP III: National Cholesterol Education Program‑Adult Treatment Panel III; No.: number ;NR: not reported; OR: odds ratio; POMS: Profile of Mood States; RR: relative risk; SAS: Self-Rating Anxiety Scale; SD: standard deviation ;SJLIFE: St Jude Lifetime Cohort; WHO: World Health Organization. a: The adjusted RR (95% CI) for WHO clinical criteria of Mets. b: The adjusted RR (95% CI) for NCEP-ATP III criteria of Mets. c: The adjusted RR (95% CI) for the IDF clinical criteria of Mets.

**Supplementary Table 4.** Quality assessment of cross-sectional studies included in the meta-analysis

| **Author, year** | **Define the source of information (survey)** | **List inclusion and exclusion criteria for exposed and unexposed subjects or refer to previous publications** | **Indicate time period used for identify inpatients** | **Indicate whether or not subjects were consecutive if not population-based** | **Indicate if evaluators of subjective components of study were masked toother aspects of the status of the participants** | **Describe any assessments undertaken for quality assurance purposes (e.g., test/retest of primary outcome measurements)** | **Explain any patient exclusions from analysis** | **Describe how confounding was assessed and/or controlled** | **If applicable, explain how missing data were handled in the analysis** | **Summarize patient response rates and completeness of data collection** | **Clarify what follow-up, if any, was expected and the percentage of patients for which incomplete data or follow-up was obtained** | **Total score** |
| --- | --- | --- | --- | --- | --- | --- | --- | --- | --- | --- | --- | --- |
| Peltzer & Pengpid, 2018 | yes | yes | yes | unclear | no | yes | no | yes | no | no | no | 5 |
| Mattei et al., 2018 | yes | yes | yes | yes | no | yes | yes | yes | no | yes | no | 8 |
| Akbari et al., 2017 | yes | yes | yes | no | no | yes | yes | yes | no | no | no | 6 |
| Moreira et al., 2019 | yes | yes | yes | no | no | yes | yes | yes | no | yes | no | 7 |
| R. cen Li et al., 2020 | yes | yes | yes | no | no | yes | yes | yes | no | no | no | 6 |
| Bagherniya et al., 2017 | yes | yes | no | no | no | yes | no | yes | no | no | no | 4 |
| Rioli et al., 2019 | yes | yes | yes | no | no | yes | yes | yes | no | yes | no | 7 |
| Kahl et al., 2015 | yes | yes | no | yes | no | yes | no | yes | no | yes | no | 6 |
| Butnoriene et al., 2014 | yes | yes | yes | no | no | yes | yes | yes | no | yes | yes | 8 |
| Roohafza et al., 2012 | yes | yes | no | no | no | yes | yes | yes | no | yes | yes | 7 |
| Tziallas et al., 2011 | yes | yes | no | yes | no | yes | no | yes | no | no | no | 5 |
| Cohen et al., 2010 | yes | yes | yes | yes | no | yes | yes | yes | no | no | no | 7 |
| Van Reedt Dortland et al., 2010 | yes | yes | no | yes | no | yes | no | yes | no | no | no | 5 |
| Carroll et al., 2009 | yes | yes | no | no | no | yes | yes | yes | no | yes | yes | 7 |
| Hildrum et al., 2009 | yes | yes | yes | no | no | yes | yes | yes | no | no | no | 6 |
| Takeuchi et al., 2009 | yes | yes | no | no | no | yes | yes | yes | yes | yes | yes | 8 |
| Vogelzangs et al., 2007 | yes | yes | no | yes | no | yes | yes | yes | yes | yes | yes | 9 |
| Skilton et al., 2007 | yes | yes | no | yes | no | yes | yes | yes | yes | yes | yes | 9 |
| Herva et al., 2006 | yes | yes | no | yes | no | yes | yes | yes | no | yes | no | 7 |
| Luppino et al., 2011 | yes | yes | no | yes | no | yes | yes | yes | yes | yes | yes | 9 |
| Albert et al., 2013 | yes | yes | no | yes | no | yes | yes | no | yes | yes | no | 7 |
| Tziallas et al., 2012 | yes | yes | no | yes | no | yes | yes | yes | no | no | no | 6 |
| Glaus et al., 2013 | yes | yes | no | yes | no | yes | no | yes | no | no | no | 5 |
| Berto et al., 2022 | yes | yes | yes | yes | no | yes | no | yes | no | no | no | 6 |

**Supplementary Table 5.** Quality assessment of cohort studies included in the meta-analysis

| **Author, year** | **Selection** | | | |  | | **Comparability** |  | **Outcome** | | | **Total score** |
| --- | --- | --- | --- | --- | --- | --- | --- | --- | --- | --- | --- | --- |
|  | **Representativeness of the exposed cohort (⋆)** | **Selection of the non-exposed cohort (⋆)** | **Ascertainment of exposure (⋆)** | **Outcome not present at start of study (⋆)** | |  | **Comparability of cohorts on the basis of the design or analysis (⋆⋆)** |  | **Assessment of outcome (⋆)** | **Follow-up long enough for outcomes to occur (⋆)** | **Adequacy of follow-up of cohorts (⋆)** |  |
| Lubas et al., 2021 | 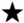 | 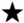 | 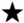 | 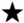 |  | | 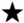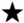 |  | 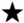 | - | - | 7 |
| Räikköonen et al., 2007 | 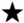 | - | 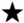 | 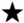 |  | | 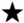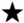 |  | 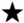 | 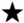 | - | 7 |
| Takeuchi et al., 2009 | 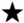 | - | 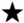 | 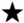 |  | | 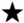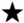 |  | 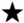 | - | - | 6 |
